# Effects of repeated freeze and thaw cycles on the stability of faecal microbiome composition

**DOI:** 10.1038/s41598-026-39939-w

**Published:** 2026-02-19

**Authors:** Matteo Sangermani, Indri Desiati, Nicole Quattrini, Guro F. Giskeødegård

**Affiliations:** 1 HUNT Center for Molecular and Clinical Epidemiology, Department of Public Health and Nursing, Norwegian University of Science and Technology, Trondheim, Norway; 2 Department of Surgery, St. Olavs University Hospital, Trondheim, Norway; 3 Department of Circulation and Medical Imaging, Norwegian University of Science and Technology, Trondheim, Norway

**Keywords:** Microbiome, Faeces, Sample handling, Freezing, 16S rRNA gene sequencing, Compositional stability, Reproducibility, Biological techniques, Microbiology

## Abstract

Reanalysing previously collected samples to address new research questions offers a time-efficient alternative to initiating new cohort studies. However, concerns remain regarding the stability of the faecal microbiome following repeated freeze–thaw (FT) cycles, which may introduce bias in downstream analyses. To this end, we investigated the effect repeated FT cycles had on 16 S rRNA gene sequencing and its ability to accurately measure the gut microbiome composition consistently. Our analysis showed that inter-individual sample differences consistently outweighed any effects introduced by FT. Differential abundance analysis with MaAsLin2 identified a limited number of significantly altered microbial genera after the first FT, when compared to the fresh, unfrozen material. The following second and third FT cycles showed no significant changes compared to the first FT cycle. Subsequent cycles (four to six) showed minor but progressive shifts in specific taxa. However, analysis with the more conservative method ALDEx2 showed no significant changes across any FT cycle. These findings support the reuse of stored faecal samples that have undergone a single thaw, suggesting minimal risk of compromising microbiome integrity.

**Supplementary Information:**

The online version contains supplementary material available at 10.1038/s41598-026-39939-w.

## Introduction

The gut microbiome is an important element of the host’s health and homeostasis. Most research on the gut microbiome collects faecal samples to explore the colonic microbial ecosystem. The colon is the section most densely colonized by microbes and the site where many of the most important microbial-host interactions take place. Collection and analysis of faeces is relatively straightforward; however, sample collection and storage practices may affect the reliability and reproducibility of microbiome analysis^1^. Factors such as transportation, storage method, temperature, and repeated thawing cycles can affect DNA extraction efficiency and alter the microbiome composition such that the sample becomes less representative of its original state.

Ideally, faecal samples would be analysed right after defecation. However, in the context of population studies and clinical trials this is not feasible because samples are usually collected by participants at their home. Thus, there is a delay after defecation in which the samples are commonly stored at room temperature (RT) or at 4 °C for several hours or a few days, before being frozen for long-term storage. This delay may alter the state of the faecal microbial and metabolic composition, but there are contrasting results on how post-defecation time impact the composition of faecal samples. A past study found systematic changes in the microbiome composition based both on the type of collection method and time window before deep freezing; however, differences between individuals generally accounted for the greatest portion of variability between samples^2^. On the other hand, a recent study found only minor changes in composition of samples kept at RT for 4 days compared to samples frozen after 30 min of defecation and fresh samples^3^.

Upon receiving the faecal samples, a common practice is to homogenize them before freezing or analysis, which fosters low within-sample variability. However, studies that subsampled different regions of faeces did not find significant differences in the outcome of compositional analysis, and in these cases the effect of individual variability was greater than variability between regions^4,5^.

In the context of clinical studies, faecal samples are usually frozen for long-term storage before further analysis. The most common storage methods are freezing, snap freezing, or lyophilization. A recent study explored different storage methods for the gut and oral microbiome, comparing fresh, lyophilized, frozen, and faecal samples stored at RT for 72 h^6^. Of these, lyophilization affected the composition of microbial communities the most, whereas the other storage methods did not significantly change the composition of microbial communities in the faecal samples. In line with these results, a previous study found no significant difference in the microbiome composition of faecal samples that were snap-frozen compared to samples that were simply frozen by placing them in the freezer^7^.

Studies have also explored if storage temperature or storage time may bias microbiome composition of faecal samples. An early study compared two-week storage at either − 80 °C–− 20 °C and did not find significant changes in composition and diversity^8^. Longer freezing periods, to as much as 6-month storage at − 80 °C, also did not affect the microbiota and the composition displayed high similarity to fresh samples^9^. Furthermore, a study found no significant changes in microbiome composition even after two years of storage^10^. Overall, past studies show that that faecal samples stored at − 80 °C preserve the original microbial composition well. This raises the possibility to reanalyse past sample cohorts for new studies and to combine different cohort for re-sequencing using improved technologies.

Repeated freeze-thaw (FT) cycles may introduce distortion and bias the microbiome composition from its original state. In the process of freezing and thawing, microbial cells undergo physical stress from ice crystals and osmosis^11^. Few studies have investigated the effect of multiple rounds of freezing and thawing of faecal samples for microbiome studies. A past study on sputum samples showed significant distortion in the composition after repeated FT cycles (thawed for 30 min per cycle)^12^. For faecal samples, a previous study showed that freezing and thawing had an effect only after the 4th cycle^13^ but changes in the microbiome composition were minor. However, this study analysed the microbiome via qPCR which limited analysis to just six higher taxonomic ranks of bacteria. Moreover, samples were manually ground to a fine powder in liquid nitrogen and aliquoted before storage at − 80 °C. This practice is often impractical in the context of large-scale clinical studies, and these findings cannot be extended to the context of most faecal sample collections.

There have been a few other studies on the effect of repeated FT cycles, but on infants or non-human samples^10,14^. For example, one past study explored the effect of multiple FT cycles on the community composition of poultry, and observed significant changes after one FT cycle^15^. Findings on storage and FT effect from these samples cannot be safely extended to adult humans because the physical properties as well as the microbial species are different. In summary, past studies have limited applicability in the context of the present practices and how faecal samples for clinical studies are collected due to sample origin or methodology employed for analysis^3,5^.

Given the gap in knowledge on the effect of repeated FT cycles we aimed to investigate the effect of repeated FT cycles on microbiome compositional analysis of human faecal samples. For this exploratory study, we employed a cohort of five individuals whose fecal sample were frozen and thawed six times. We optimized a process of sample thawing to limit unnecessary, prolonged thawing time and determined the effect of repeated FT cycle by examining differential abundance of taxa and inter-individual variations of 16s rRNA gene sequencing of the fecal samples.

## Materials and methods

### Cohort and sample collection

We recruited five individuals who provided faecal samples. The study was performed in accordance with relevant guidelines, and informed consent was obtained from all participants. Participants were healthy and between the ages of 25 and 50 years; they did not report intestinal problems or disorders; and did not undergo antibiotic treatment during the sampling period. Sample collection was performed by the individuals at their home, using the following procedure: faecal samples were collected placing the Fe-Col faecal catcher (Fe-Col, cat: FC2010, AlphaLaboratories, UK) on the toilette seat and a subsample was transferred into sterile 15 mL cryogenic vials (cat 479-0006, Nalgene, ThermoFisher). Volunteers were tasked to fill about one third of the cryogenic vial with faecal matter, which corresponded to about 6–8 g per vial. The samples were then stored at ~ 4 °C in home refrigerator and delivered for storage within 12 h. To assess the reproducibility of the methods additional control samples were prepared from one individual that was not included in the original study cohort. In addition, three individuals provided faecal samples used to optimize the thawing process.

### Sample storage, freezing and thawing procedure

Upon receiving the vial from a volunteer, faecal matter was homogenised by manual mixing. We then took a subsample of freshly delivered subsample (i.e., never frozen), about 180 mg of the freshly mixed material, and placed in a 2 mL Eppendorf tube for immediate DNA extraction. This DNA extraction provided the baseline microbiome composition of the faecal sample (labelled C0 in this study). The remaining faecal sample in the 15 mL cryogenic vials was stored at − 80 °C until further subsampling and analyses.

To investigate the effect of repeated cycles of freezing and thawing, we performed the following procedure every three to four days (for a total of six times) for each individual in our cohort: the vials containing faecal samples were taken from − 80 °C and placed at − 20 °C for 20 min; then the vials were moved to + 4 °C for 20 min. We then took a subsample (180 mg) and placed it in a 2 mL Eppendorf tube for DNA extraction. Subsampling was performed using a sterile hard-plastic spatula. Faecal material remained solid when subsampling and we used the spatula to apply pressure to chip away faecal material. Subsampling created crumbles and faecal material did not display wetness or malleability at this stage. Right after subsampling, the remaining faecal sample in the 15 mL cryogenic vials was placed back at − 80 °C.

### Thawing processes and temperature measurements

Three individuals collected faecal samples that we used to measure the changes in temperature of faecal samples during thawing. These individuals collected faecal material in 3 cryogenic vials (following the same collection protocol as described above). Upon receiving the samples in the lab, we split the faecal samples in differently sized cryotubes of 2 mL, 5 mL, and 15 mL, with each having 1.5 g, 4 g, and 8 g of faeces, respectively. Samples were then stored at − 80 °C for one week.

We designed and tested two thawing processes: one fast and one slow. In the slow thawing the tubes were taken from − 80 °C and placed for 20 min at − 25 °C, then moved for 20 min at + 4 °C, and then for 30 min at room temperature (RT). In the fast-thawing design samples were taken from − 80 °C and placed at RT for 60 min. Temperature was regularly recorded every 5 min during the thawing process, using a 62 MAX Mini Infrared Thermometer (Fluke, US). We performed each thawing condition (fast or slow) and in each tube type (2 mL, 5 mL, and 15 mL) for *n* = 3 individuals (biological replicas), each measured in 3 technical replicas.

### DNA sequencing, library construction and sequencing

Each subsampling was performed in a sterile biosafety cabinet, but without active airflow. Using QIAamp PowerFaecal Pro DNA kit (cat: 51804, Qiagen) we performed extraction of DNA using 180 mg of faecal material as input. The extracted, template DNA was eluted using nuclease-free water (W4502, Sigma-Aldrich), and stored at − 20 °C in low binding Eppendorf tubes (0030108051, Eppendorf). DNA samples were quantified by Qubit HS-DNA assay (Thermo Fisher). Extracted DNA was normalized to 1 ng/µL before using it as template for library preparation. Libraries were created using the QIAseq 16 S/ITS Region Panel kit (333842, Qiagen), which allows to target and amplify multiple regions of the 16 S rRNA gene at the same time. We targeted regions V1V2 and V5V7 (target areas of primers are approximately 350 nt and 410 nt, respectively). After amplification samples were normalized to a concentration of 14 ng/µL and multiplexed to sequence all samples in the same batch, using QIAseq 16 S/ITS Index kit, set A (333822, Qiagen). Pair-ended sequencing (2 × 300 bp) was performed using the MiSeq V3 flowcell platform (Illumina). Combined with the QIAseq 16 S/ITS Region Panel kit, which uses phased primers, we obtained around 20–22 M raw reads per flowcell after filtering PhiX spacer reads. Sequencing was performed at the Genomics Core Facility, NTNU.

### Bioinformatics for sequencing data

We created our pipeline to analyse raw sequences data using QIIME2^16^. Specifically, we screened for potential human contaminants with Bowtie2 v.2.3.036 and employed DADA2 as denoising algorithm to merge and quality filter sequences^17^. ASVs were assigned a taxonomic nomenclature using a classifier built from the reference database SILVA (version 138.1). The parameters for the denoise algorithm were the following: no trimming (--p-trim-left-f and -r set at 0), low-quality bases were truncated at 240 nt for forward reads (--p-trunc-len-f 240) and at 200 nt for reverse (--p-trunc-len-r 200), and we set maximum expected errors for forward reads at 6 (--p-max-ee-f 6) and at 8 for reverse (--p-max-ee-r 8). The taxonomic features from the two individual regions (V1V2 and V5V7) were used as a combined dataset. We obtained on average 125 000 reads per sample, and ~ 95% of reads passed decontamination and quality-trimming steps for downstream analysis (the rest were discarded from further analysis). Sufficient sequencing depth was confirmed by examining rarefaction curve and a plateau was reached at around 30 000 reads. Of the initial 5327 ASVs, we removed those that had an average relative abundance of less than 0.03% in the cohort and if present in more than 10% of all samples, leaving in total 2540 unique ASVs for the entire cohort, which accounted for a total of 180 genera, from 56 families, and 18 classes.

### Data analysis

We performed all analyses and visualization using the R language^18^. We used packages tidyverse (1.3.2), dplyr (1.0.10), stringr (1.5.0) for data handling, and results were visualized using ggplot2 (3.4.0)^19^. We used vegan (2.6-4) to calculated alpha diversity (measuring within-sample diversity, including Shannon effective number (expected) and taxa richness) and beta diversity metrics (measuring differences in diversity between samples, including Bray-Curtis, Jaccard, and Aitchison (robust)). Test statistics were conducted using the package rstatix (0.7.2). To compare differences in diversity metrics of FT cycles we run a linear mixed-effect model using individuals ID as random effect and FT cycle as fixed effect, and post-hoc pairwise comparisons using estimated marginal means (EMMs) with FDR Benjamini-Hochberg adjustment. The coefficient of variation (CoV) and interclass correlation coefficient (ICC) were calculated to assess the stability of individual features over time, for individual taxonomic features. For the ICC we employed the R package nlme (3.1–164) to fit linear mixed-effects models from which we extrapolated the ICC values. Principal component analysis (PCA) was performed for dimensional scaling and data clustering visualization, using the R package mixOmics (6.24.0)^20^. Moreover, we performed ordination analysis via non-Euclidean dimensional reduction of beta-diversity distance matrices using the algorithm PCoA and NMDS, using Bray-Curtis dissimilarity as input, which in turn was generated from centre log-ratio transformed features. We performed PERMANOVA tests on the ordination analysis to measure statistical differences between groups, via adonis2 function of the R package vegan (2.6-4). The t-distributed stochastic neighbour embedding (tSNE) algorithm was performed to visualize the within- and between-individual variation using R package Rtsne (0.16). The features (i.e., genera) were mean centred and scaled before performing tSNE with a perplex parameter set to 10.

We used several methods to perform differential abundance analysis and identify compositional changes in individual features of the microbiome. First, we employed univariate analysis of each genus via Wilcoxon signed rank between FT cycle C0 and C6. Secondly, we then used two methods that use generalized linear models to analyse all FT cycles simultaneously: ANOVA-Like Differential Expression analysis (ALDEx2) using the R packages ALDEx2 (1.36.0)^21^ and MaAsLin2 (Microbiome Multivariable Association with Linear Models) via the R packages MaAsLin2 (1.18.0)^22^. In both generalized linear models of ALDEx2 and MaAsLin2 we used the FT cycles as fixed effect and corrected for the random effect of individual ID. In addition, to limit false discovery rate we followed establish practices^23^. In ALDEx2 the read-count feature table was centre-log ratio transformed, whereas in MaAsLin2 the read-count feature table was normalized via total sum scaling and log2-transformed. In all differential abundance analysis, we adjusted p-values for multiple testing using Benjamini-Hochberg method.

## Results

### Thawing rate of faecal samples

Thawing is necessary before subsampling frozen faecal samples and thawing speed is dependent on the size of the sample. To optimize and ensure a consistent outcome of the thawing process, we measured faecal samples stored in cryotubes of 2 mL, 5 mL, and 15 mL, containing 1.5 g, 4 g, and 8 g of faeces, respectively. We performed the thawing process following two approaches: a fast-thawing and a slow-thawing approach (Suppl. Figure 1). Slow thawing using 15 mL tubes, with approximately 8 g of faeces was the most gradual temperature transition approach (Suppl. Figure 1B). This maintained samples at around 0 °C for extended period of time, maintaining most microbes at a dormant state, which minimize their growth. Moreover, this approach allowed an extended time window to process in parallel multiple samples, thereby guaranteeing we could repeat FT cycles with systematic and consistent experimental conditions, namely temperature. Therefore, we used this slow-thawing design with samples collected in 15 mL tubes for the following experiments on the effect of repeated freezing and thawing.

### Gut microbial diversity over FT cycles

**Fig. 1:**
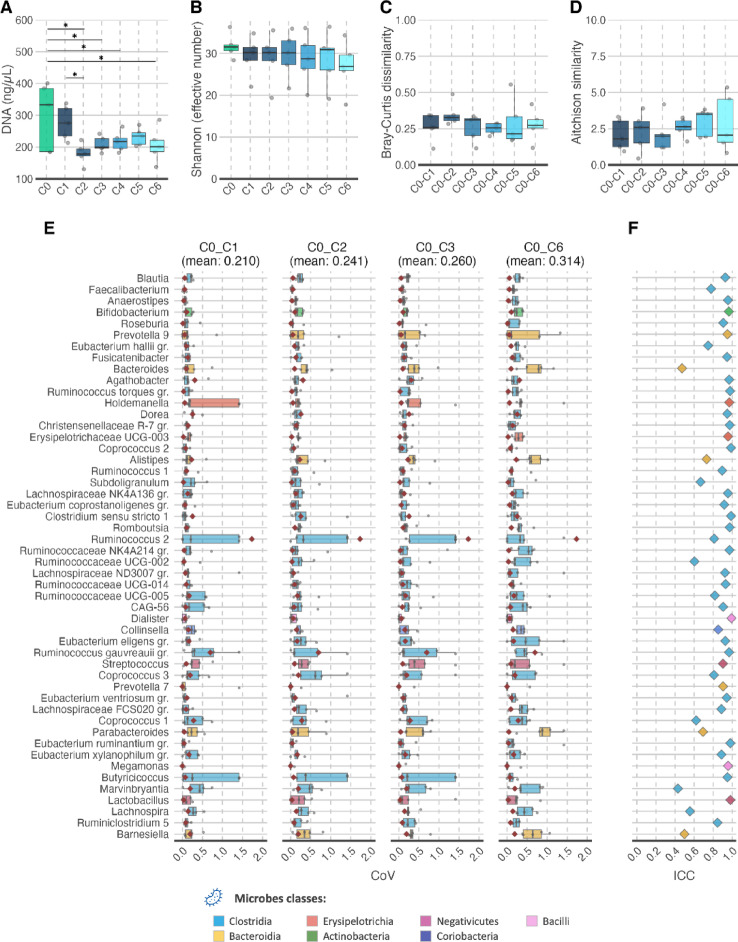
Assessing changes in diversity and features variability during repeated freezing thaw cycles. (**A**) Concentration of extracted DNA from faecal samples at different FT cycles. (**B**–**D**) Diversity metrics measured at different thawing cycles: alpha-diversity Shannon effective number (**B**), beta-diversity metrics Bray-Curtis dissimilarity (**C**) and Aitchison similarities (**D**). In A to B, x-axis groups shown individual time points (**A** and **B**) or comparing timepoint C0 (fresh sample) to each of the following thawing cycle, i.e., C1 to C6 (**C** and **D**). (**E**) Coefficient of variation (CoV) for the top 50 most abundant genera (sorted by abundance) in the gut microbiome of the entire cohort, comparing FT cycle C0 to C1, C2, C3 and the C6 (C4 and C5 in Suppl. Figure 2 C). The red dots are control values, CoV calculated from set of quality control samples (*n* ≥ 3). (**F**) Interclass correlation (ICC) of the top 50 most abundant genera, sorted by abundance (as shown in y-axis of chart (**E**)). Box plot – line, mean; box, delimits 25th and 75th percentile; whiskers, 1.5 interquartile; points, individual values. Also, in E and F boxes are coloured according to the features hierarchical grouping (see legend).

To determine the effect of repeated freezing and thawing on faecal samples, we analysed faecal samples from a group of 5 healthy volunteers: fresh faeces, i.e., never frozen samples (labelled as C0 in Fig. 1); and after each of six FT cycles (labelled as C1- C6 in Fig. 1). We observed significant changes in DNA concentration associated to FT cycles (linear mixed-effect model, *p* < 0.01). Post-hoc pairwise comparisons revealed differences between unfrozen sample (C0) and C2, C3, C4 and C6 cycles (p_*adj*_ < 0.05; Fig. 1A). The subsequent FT cycles did not display significant change in DNA concentration, except between C1 and C2. The alpha diversity metrics Shannon and richness did not show statistically significant changes across FT cycles (LMM: *p* > 0.05; Fig. 1B and Suppl. Figure 2 A). Analysis of the beta diversity metrics Bray Curtis, Aitchison and Jaccard– using C0 as reference time point –were unaffected as well by the repeated cycles of freezing and thawing (LMM: *p* > 0.05; Fig. 1C and D and Suppl. Figure 2B).

### High reproducibility maintained after multiple FT-cycles

We calculated the coefficient of variation (CoV) and intraclass correlation coefficient (ICC) to assess the stability of features during multiple FT cycles. The CoV remained largely stable in most microbial genera when comparing C0 with other FT cycles (Fig. 1E and Suppl. Figure 2 C). Moreover, in most taxa the distribution of CoVs were close to their respective CoV for repeated measurements (red point in Fig. 1E, ranging on average at 11%). However, there were genera that had increasing CoV values with increasing FT cycles. Notably genera *Bacteroides*, *Alistipes*, *Parabacteroides*, *Barnesiella*, and to a lesser extend *Prevotella* (all belonging to class Bacteroidia) had increased mean CoV values at later FT cycles. There were also a few genera belonging to family Ruminococcacea and Lachnospiracea (belonging to class Clostridia) which showed increasing CoV values with increasing FT cycles, albeit to a smaller degree than the class Bacteroidia (Fig. 1E, Suppl. Figure 3 A and 2 C).

The ICC values were higher than 0.8 for 39 of the top 50 genera, which signify a low variance between FT measurements, i.e., high reliability and reproducibility of repeated measurements (Fig. 1F). *Faecalibacterium*, *Eubacterium halii*, *Alistipes*, *Subdoligranulum*and Parabacteroides had ICC values ranging between 0.6 and 0.8, indicating that these features are more variable during repeated FT cycles, albeit modestly. The genera *Bacteroides*, *Ruminococcus UCG-002*, *Coprococcus*, *Marvinbryantia*, *Lachnospiria* and *Barnesiella* had ICC values under 0.6, indicating higher variance between FT cycles. These results were consistent when examining changes at higher taxonomic ranks, such as class and family (Suppl. Figure 3B and 3D). We observed that all families belonging to class Bacteroidia had ICC values below 0.8, and their CoV increased over FT cycles; families Bacteroidacea and Barnesiellaceae had especially low ICC values. The family Lachnospiraceae (of class Clostridia) also had lower ICC values (mean 0.67; Suppl. Figure 3D). The classes Gamma-, Delta- and Alphaproteobacteria, had wide interquartile range in their CoV distributions and low ICC values, indicating that they may be influenced by repeated FT cycles. These latter classes were present in small quantities and not detected in all individuals tested.

**Fig. 2:**
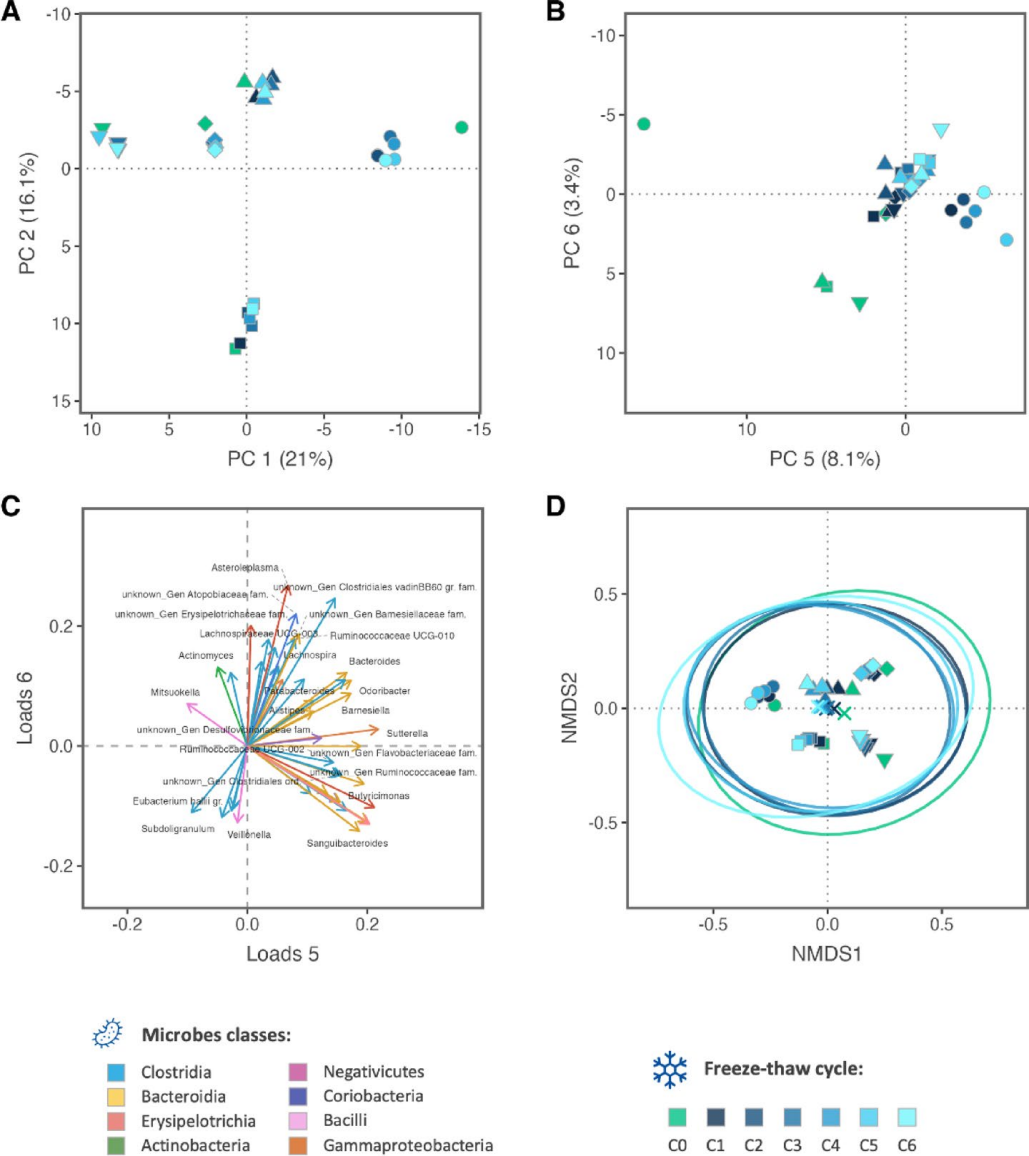
Multidimensional analysis of the microbiome and the effect of freezing and thawing. A and B – Principal component analysis (PCA) scores for the first two components (**A**) and for the 5th and 6th components (**B**). In parenthesis is shown the variance explained by each component. (**C**) Loadings of the components 5 and 6 from the PCA. Arrows colours indicate the class or origin of each genus (see lower left legend). (**D**) NMDS with Bray-Curtis dissimilarity, PERMANOVA test showed no difference between FT cycles (*p* > 0.05). In A, B and C shape of points indicate different biological replicas (individuals), whereas of points and circle colour indicate different FT cycles (see lower right legend).

### Intra-individual differences are much larger than differences due to FT cycles

To visualize multivariate patterns from FT cycles using all the microbial features we performed multidimensional reduction analysis. In the PCA, the scores PC5 and PC6 (which together explained 11.5% of the variance) showed a tendency of separating samples according to the FT, with the main separation being between unfrozen (C0) and frozen samples (C1 to C6) (Fig. 2B). However, in the first 4 PCs (which explained 84.6% of the variance) the sample clustering was dominated by inter-individual differences, as shown by the score plot of PC1 and PC2 (Fig. 2A, loadings in Suppl. Figure 4 C), as well as PC3 and PC4 (Suppl. Figure 4B). In the loadings of PC5 and PC6 the genera *Odoribacter*, *Bacteroides*, *Alistipes*, *Barnesiella* (mainly from class Bacteroidia) and *Sutterella* (from class Gammaproteobacteria) are amongst the greatest contributors for the separation of samples by FT cycle (Fig. 2C). The tSNE analysing showed clustering by individuals rather than FT cycles (Suppl. Figure 4 A).

To further investigate multivariate effects, we performed ordination by non-metric dimensional scaling (NMDS) and PCoA (Fig. 2D and Suppl. Figure 4D). These also demonstrate that samples clustered by individual. In the NMDS we observe a consistent but very minor shift in centroids that follows the freezing and thawing cycles sequence. PERMANOVA test showed no significant difference between the different FT cycles (*p* = 0.482).

### Taxa of class bacteroidia are most affected by freezing and thawing

To further determine the effect of FT cycles on individual genera we performed Wilcoxon signed rank test between freezing timepoint C0 and C6. No feature was found significantly different after multiple testing adjustment (*p* > 0.05) (Suppl. Table 1).

**Fig. 3:**
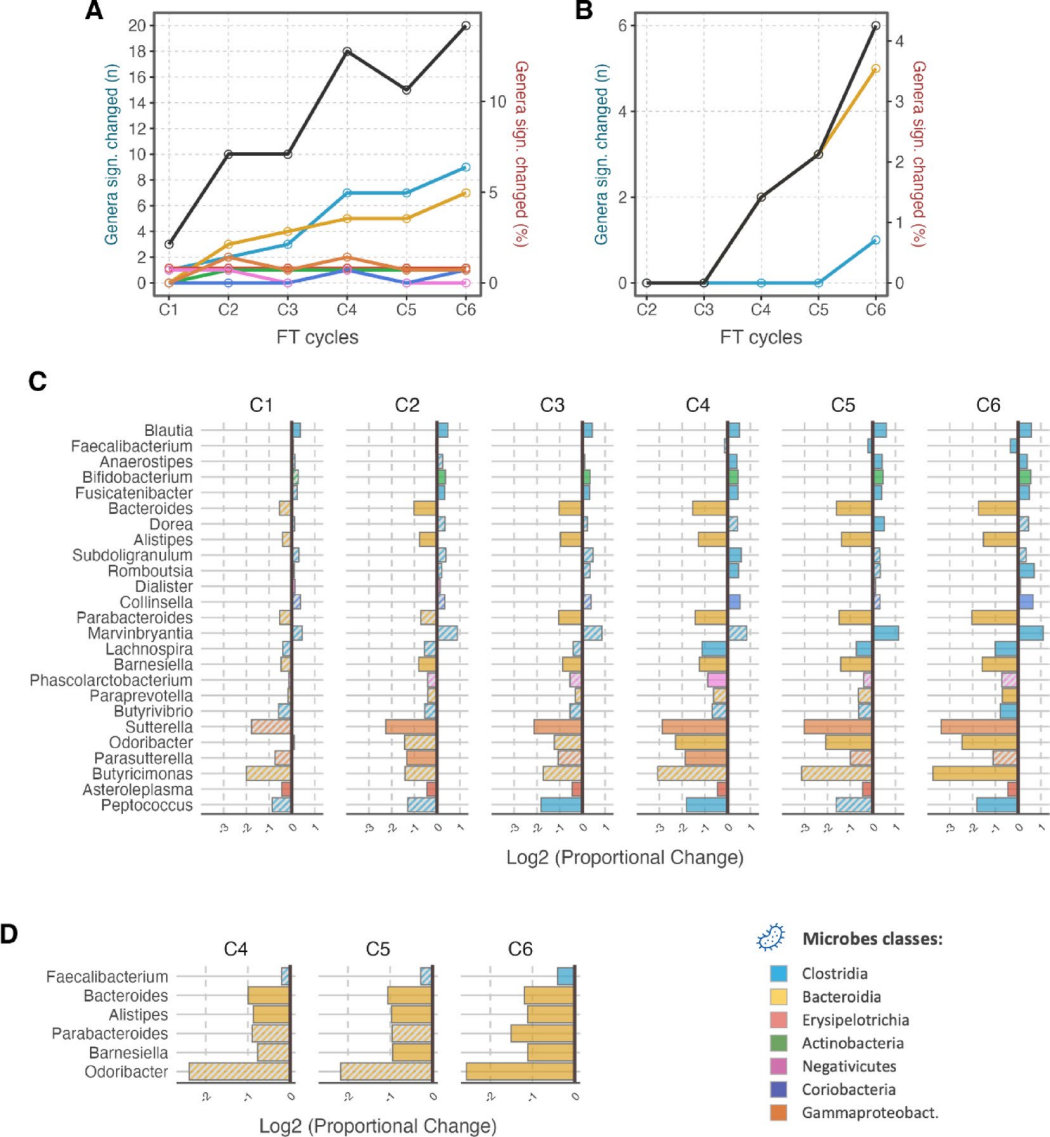
Genera of the class Bacteroidia are prominently affected by repeated FT cycles. (**A**) and (**B**) The line plots show the number of genera that are significant changes at each FT cycle point with respect to the reference timepoint: this is C0 (never-frozen sample) in (**A**) and it is C1 in (**B**). Each line shows the number of genera in a specific Class that were found to have a significantly changes abundance. The black line tracks the total number of genera. The right y-axis displays the proportion of total number of genera that were significantly changed. C and D – The plots chart the log2-fold change in the abundance of genera at different FT cycles. In (**C**) the reference timepoint is C0, whereas in (**D**) the reference timepoint is C1. In (**D**) the FT cycles C2 and C3 are not shown because no significant feature was found. Statistically significant changes are shown as solidly coloured bars (*p* < 0.05); statistically insignificant changes (*p* > 0.05) are shown as bars with a striped pattern. Genera in the y-axis are sorted by their average abundance in the cohort.

We then employed linear modelling to evaluate the association of microbial features to repeated FT cycles. Two differential analysis tools, ALDEx2 and MaAsLin2, were employed and they handle the complexity and peculiarity of the microbiome data in the context of population studies. ALDEx2 is considered a more conservative method in its evaluation of differential abundance, whereas MaAsLin2 is more sensitive, albeit at the expense of potential higher rate of false discovery^24^. ALDEx2 revealed no significant changes in features during any FT cycle (Suppl. Table 1). MaAsLin2 found some significant changes in the microbiome during repeated FT cycles (Fig. 3A and B).

When comparing FT cycle C1 to subsequent ones (Fig. 3B), we observe significant changes only after C4 and in only 2.5% of all genera. This increases to 4% by FT cycle C6, and these are five genera from class Bacteroidia, and one genus from class Clostridia. At C4 and C5 we observed genera *Bacteroides*, *Alistipes* and *Barnesiella* having significant decrease in abundance in respect to the baseline C1 (Fig. 3D), with an average log2-fold change of − 0.96 (SD: ±0.08), equivalent to − 48.8% decrease. By FT cycle C6 genera *Faecalibacterium* (from class Clostridia) showed a significant log2-fold change of − 0.40, equivalent to − 24.2%. By FT cycle C6, all genera of the class Bacteroidia experienced a substantial decrease in abundance (an average log2-fold change of − 1.49 (SD: ±0.61), equivalent to − 64.4%), with genus *Odoribacter* showing the largest decrease, at − 83.0%.

When comparing a fresh sample (C0) to subsequent FT cycles, there were significant changes in the microbiome from the first thawing (C1) (Fig. 3A and C). At C1, three genera underwent significant changes, *Blautia*, *Dialister* and *Asteroleplasma*, with log2-fold changes of 0.37, 0.14, − 0.44, respectively (equivalent to + 29.2%, + 10.2% and − 26.3%). In subsequent FT cycles genera from class Bacteroidia and Gammaproteobacteria decreased consistently. Genera of class Bacteroidia experienced by C4 average log2-fold changes of − 0.97 (SD: ±0.08) (equivalent to − 48.9%) and by C6 of − 1.97 (SD: ±0.95) (equivalent to − 74.5%). Between C4 to C6, genera of class Gammaproteobacteria had on average log2-fold changes of − 2.79 (SD: ±0.65) (equivalent to − 85.5%). In contrast, the abundance increased over repeated FT cycles in genera *Blautia*, *Anaerostipes*, and *Fusicatenibacter* (of class Clostridia), *Bifidobacterium* (of class Actinobacteria) and *Collinsella* (of class Coriobacteria), with an overall average log2-fold change of 0.54 at C6 (SD: ±0.09) (equivalent to + 45.4%). Some very low abundance genera displayed greater significant changes. For example, the log2-fold changes by C6 were 0.71 for *Romboutsia* (equivalent to + 63.6%) and 1.10 for *Marvinbryantia* (equivalent to + 114.3%), both belonging to class Clostridia. A notable exception to the positive association of genera from class Clostridia with FT cycles, were *Faecalibacterium* and *Peptococcus*, which displayed log2-fold changes of − 0.33 and − 1.81, respectively (equivalent to -20.4% and − 71.5%).

Overall, we observed that some classes had a larger portion of genera associated with FT cycles when comparing to the unfrozen samples: 11 (equivalent to 11.8%) from class Clostridia; 7 (39%) from class Bacteroidia; two (25%) from class Negativicutes and Gammaproteobacteria each; one (25%) from class Erysipelotrichia; and one from classes Coriobacteria and Actinobacteria each (8.3% and 9.1%, respectively).

## Discussion

In this study, we investigated the impact of repeated freezing and thawing cycles on the microbial composition of faecal samples. Previous studies on this subject have been conducted on non-human samples, or infants, or employed methods that are today considered limiting, or have used labour intensive storage practices (e.g., via lyophilization or grounding to powder and aliquoting). Compared to past studies, our results are applicable to the context of methodologies and research practices that are used today. Our results suggest that repeated FT cycles using a gradual thawing of faeces samples, allows to maintain a high degree of reproducibility of the gut microbiome composition when analysed by 16s rRNA gene sequencing.

To minimize the risk of DNA degradation, we monitored the DNA concentrations throughout the FT cycles. DNA concentrations were significantly reduced after one FT cycle while subsequent cycles did not show significant decrease in DNA concentration. This suggests that microbial DNA remained largely intact and sufficient to obtain reliable community analysis comparable to fresh samples.

Repeated freezing and thawing cycles did not significantly alter the alpha diversity of the gut microbial community or the beta diversity when comparing the fresh samples to the next six cycles of freezing and thawing. Principal component analysis revealed a small tendency for separation of sample related to FT cycles (in PC5 and PC6). However, inter-individual differences remained larger and predominant even after six FT cycles in all other principal components (PC1 to PC4) as well as in the ordination analysis (NMDS and PCoA). These results suggest that microbial diversity remains consistent throughout the repeated series of FT cycles and that inter-individual differences dominate over FT effects. We hypothesise that the structure of faecal material and the thawing procedure we employed may have contributed to this. Faecal material is rich in solutes and macro-molecules that may limit the mechanical damage microbes created during thawing and limit the lethal concentration of salts in the cell vicinity during FT process. This can explain why past studies on oral microbiome, a bio-sample that is scarce in solutes, found low preservation in microbial communities after repeated freezing and thawing^6^. Moreover, our approach ensures samples remained near 0 °C when sub-sampling, with faeces soft enough that a simple plastic disposable spatula and no large manual force was required to chisel away solid pieces of faecal material.

While neither ALDEx2 nor Wilcoxon rank-sum identified any significant changes after six FT cycles, differential abundance analysis using MaAsLin2 detected significant changes for some microbial features. More specifically, MaAsLin2 identified a limited number of significantly altered microbial genera between fresh, unfrozen material and samples frozen once. The following second and third FT cycles showed no significant changes compared to the first FT cycle, while subsequent cycles showed minor but progressive shifts in specific taxa. However, these results must be taken with caution as they were not detected by more conservative methods, such as ALDEx2. Recent studies have highlighted the caution necessary to use appropriate methods to investigate differential abundance of microbial features in different sample groups. One recent article highlighted how past studies have employed methodologies that are often prone to false positives findings in microbiome studies^25^. This was confirmed by another methodological study, that compared several differential abundance analysis methods and found some are prone to a disturbing high rate of false discoveries^24^. In our studies, we employed multiple methods to find consensus and increase robustness of results. Thus, given the lack of consensus with two other methods, the significant changes we identified by MaAsLin2 can represent false positive results.

MaAsLin2 findings of microbial genera associated with changes during FT cycles were notable for showing good alignment between the genera and their average gut microbiome abundance, as well as Gram staining. Our previous longitudinal study that analysed first-time thawed samples, reported that genera such as *Collinsella* and *Marvinbryantia* (of class Coriobacteria and Clostridia, respectively), and *Parabacteroides*, *Barnesiella* and *Odoribacter* (of class Bacteroidia) each constitute on average less than 0.5% of the gut microbiome^26^. These genera showed the lowest stability during repeated FT cycles. On the other hand, more abundant genera, such as *Blautia* and *Faecalibacterium* (about 8% each), *Bacteroides* and *Alistipes* (about 4% and 1.5%) displayed significant but smaller effects due to FT cycles.

We noticed that Bacteroidia and Proteobacteria are classified as Gram-negative bacteria, whereas Clostridia and Actinobacteria largely stain as Gram-positive. It could be interesting to investigate in future if the thickness of the peptidoglycan layer, which partly overlap with Gram staining classification, contribute to the resilience to physical stresses posed by freezing and increased preservation in the fecal sample. *Prevotella 9* genus (which constitute on average 4% of the gut microbiome), despite being a Gram Negative of class Bacteroidia, was not found to be significantly changed. *Prevotella* is notable for being associated with polysaccharide use^27^. This lead to the interesting, but yet untested hypothesis, that physical proximity to hygroscopic vegetable fibres present within faeces might help shield gut microbes from the worst effect of freezing and thawing, thus, creating a preservation bias of bacteria that digest fibers.

The sample size of this exploratory study was small and consisted exclusively of healthy adult individuals, which is a limitation. A more heterogeneous cohort, including individuals of varying ages, health statuses, or environmental exposures, might reveal taxa that are more sensitive to freeze-thaw (FT) cycles. Furthermore, we employed 16 S rRNA gene sequencing, which limits taxonomic resolution to the genus level. It is possible that shotgun metagenomic sequencing, which enables species-level identification, could yield different insights into microbial sensitivity of lower taxa to FT cycles. The choice of sequencing regions in 16 S rRNA gene sequencing studies can influence the taxa detected. However, we sequenced two portions of the16S rRNA gene (V1V2 and V5V7), which provide broader taxonomic coverage than using a single region. A previous study showed that the region V1V3, which overlaps with our chosen regions, offers a reasonable approximation of human gut microbial diversity^28^. Furthermore, normalization of microbiome data is a necessary step that may influence downstream analyses. We applied CLR transformation for most analyses, which is commonly used in compositional data analysis. For MaAsLin2, we used total sum scaling (TSS) normalization, as recommended. Nonetheless, different normalization techniques may yield varying results, and this remains a general challenge in microbiome research.

In conclusion, our results suggest that fecal samples subjected to a limited number of freeze-thaw cycles can be reliably reanalysed, as inter-individual variations in composition remains largely preserved. No significant changes were observed when comparing samples thawed for the first time to those thawed up to three times. However, progressive alterations in composition after the fourth FT cycle suggest reduced fidelity with further reuse. While minor changes may occur after the first time freezing, analysing fresh sample is often impractical, and faeces samples are usually collected and frozen long-term before being analysed. Our results support the feasibility of reusing previously thawed samples for repeated measurements. This opens opportunities to leverage existing sample collections to address new research questions in gut microbiome studies.

## Supplementary Information

Below is the link to the electronic supplementary material.

Supplementary Material 1

Supplementary Material 2

## Data Availability

Raw sequences are available at NCBI Sequence Read Archive (SRA), with BioProject ID PRJNA1235659 ( [https://www.ncbi.nlm.nih.gov/bioproject/PRJNA1235659](https:/www.ncbi.nlm.nih.gov/bioproject/PRJNA1235659) ).

## Code availability

Feature tables and R scripts used for analysis have been deposited and are available at the following link: (https://github.com/sanger-matteo/Freeze-Thaw-cycles).

Data availability.

Raw sequences are available at NCBI Sequence Read Archive (SRA), with BioProject ID PRJNA1235659 (https://www.ncbi.nlm.nih.gov/bioproject/PRJNA1235659).
