# Supplementary material for: Effects of repeated freeze and thaw cycles on the stability of faecal microbiome composition
Source: Sci Rep. 2026 Feb 19;16:9880. doi: 10.1038/s41598-026-39939-w (PMC13018280; doi:10.1038/s41598-026-39939-w)
Supplement: Supplementary file 1 — Supplementary Material 1 [file 41598_2026_39939_MOESM1_ESM.pdf]

Wilcoxon rank-sum tests, showing the top findings by p-value. None was found significant after adjustment

Phyla	Class	Genus	Ind_var	P.Wilcx	adj_P.Wilcx
Firmicutes	*Clostridia*	unknown_Gen Clostridiales vadinBB60 gr. fam.	FT_cycle	0,0075	0,6389
Bacteroidetes	*Bacteroidia*	Bacteroides	FT_cycle	0,0079	0,6389
Firmicutes	*Clostridia*	Faecalibacterium	FT_cycle	0,0159	0,8053
Proteobacteria	*Gammaproteobacteria*	Sutterella	FT_cycle	0,0200	0,8053
Bacteroidetes	*Bacteroidia*	Odoribacter	FT_cycle	0,0317	0,9938
Firmicutes	*Clostridia*	unknown_Gen Peptococcaceae fam.	FT_cycle	0,0449	0,9938
Firmicutes	*Clostridia*	Lachnospira	FT_cycle	0,0556	0,9938
Firmicutes	*Clostridia*	Ruminococcaceae UCG-010	FT_cycle	0,0556	0,9938
Firmicutes	*Clostridia*	unknown_Gen Clostridiales ord.	FT_cycle	0,0556	0,9938
Firmicutes	*Clostridia*	GCA-900066575	FT_cycle	0,0720	1,0000
Firmicutes	*Clostridia*	Peptococcus	FT_cycle	0,0720	1,0000
Firmicutes	*Clostridia*	Anaerosporobacter	FT_cycle	0,0776	1,0000
Bacteroidetes	*Bacteroidia*	Alistipes	FT_cycle	0,0952	1,0000
Bacteroidetes	*Bacteroidia*	Barnesiella	FT_cycle	0,0952	1,0000
Firmicutes	*Clostridia*	Lachnospiraceae UCG-003	FT_cycle	0,1231	1,0000
Proteobacteria	*Gammaproteobacteria*	Parasutterella	FT_cycle	0,1388	1,0000
Bacteroidetes	*Bacteroidia*	Parabacteroides	FT_cycle	0,1508	1,0000
Firmicutes	*Erysipelotrichia*	Asteroleplasma	FT_cycle	0,1797	1,0000
Firmicutes	*Clostridia*	Lachnospiraceae UCG-010	FT_cycle	0,1797	1,0000
Actinobacteria	*Coriobacteriia*	unknown_Gen Atopobiaceae fam.	FT_cycle	0,1797	1,0000

ALDEx2 analysis results, listing the features with the lowest p-values. P-value (top table), adjusted p-value (middle table), and effect size (bottom table). No feature was found significant after adjustment.

Phyla	Class	Genus	TestVar	Random effect	C1 p-value	C2 p-value	C3 p-value	C4 p-value	C5 p-value	C6 p-value	Intercept p-value
Bacteroidetes	*Bacteroidia*	Bacteroides	FT_cycle	(1 | Sample_ID)	0,6508	0,3011	0,2374	0,0870	0,0493	0,0343	5,19E-20
Firmicutes	*Clostridia*	Eubacterium hallii gr.	FT_cycle	(1 | Sample_ID)	0,2577	0,1015	0,1365	0,0333	0,0897	0,0414	2,32E-21
Firmicutes	*Clostridia*	Marvinbryantia	FT_cycle	(1 | Sample_ID)	0,2863	0,0639	0,0908	0,0552	0,0306	0,0307	3,10E-10
Bacteroidetes	*Bacteroidia*	Parabacteroides	FT_cycle	(1 | Sample_ID)	0,8372	0,8042	0,5592	0,4567	0,3880	0,0225	1,32E-05
Firmicutes	*Clostridia*	Ruminococcaceae UCG-003	FT_cycle	(1 | Sample_ID)	0,3915	0,1343	0,7334	0,3230	0,6547	0,0254	1,31E-02
Firmicutes	*Clostridia*	Subdoligranulum	FT_cycle	(1 | Sample_ID)	0,0584	0,0121	0,0137	0,0009	0,0226	0,0138	5,68E-24
Firmicutes	*Clostridia*	unknown_Gen Clostridiales vadinBB60 gr. fam.	FT_cycle	(1 | Sample_ID)	0,0028	0,0020	0,0021	0,0059	0,0013	0,0032	1,00E-01

Phyla	Class	Genus	TestVar	Random effect	C1 p-value adj.	C2 p-value adj.	C3 p-value adj.	C4 p-value adj.	C5 p-value adj.	C6 p-value adj.	X-Intercept p-value adj.
Bacteroidetes	*Bacteroidia*	Bacteroides	FT_cycle	(1 | Sample_ID)	1,0000	1,0000	1,0000	0,9992	0,9982	0,9195	1,87E-18
Firmicutes	*Clostridia*	Eubacterium hallii gr.	FT_cycle	(1 | Sample_ID)	1,0000	0,9985	0,9995	0,9508	0,9971	0,9670	1,09E-19
Firmicutes	*Clostridia*	Marvinbryantia	FT_cycle	(1 | Sample_ID)	1,0000	0,9979	0,9995	0,9799	0,9793	0,9643	1,98E-09
Bacteroidetes	*Bacteroidia*	Parabacteroides	FT_cycle	(1 | Sample_ID)	1,0000	1,0000	1,0000	1,0000	1,0000	0,8969	5,90E-05
Firmicutes	*Clostridia*	Ruminococcaceae UCG-003	FT_cycle	(1 | Sample_ID)	1,0000	1,0000	1,0000	1,0000	1,0000	0,7952	5,06E-02
Firmicutes	*Clostridia*	Subdoligranulum	FT_cycle	(1 | Sample_ID)	0,9888	0,8031	0,8257	0,1484	0,8960	0,8140	5,05E-22
Firmicutes	*Clostridia*	unknown_Gen Clostridiales vadinBB60 gr. fam.	FT_cycle	(1 | Sample_ID)	0,2322	0,1713	0,2077	0,3900	0,1457	0,2661	3,20E-01

Phyla	Class	Genus	TestVar	Random effect	C1 effect size	C2 effect size	C3 effect size	C4 effect size	C5 effect size	C6 effect size
Bacteroidetes	*Bacteroidia*	Bacteroides	FT_cycle	(1 | Sample_ID)	0,378	0,104	-0,006	-0,170	-0,316	-0,436
Firmicutes	*Clostridia*	Eubacterium hallii gr.	FT_cycle	(1 | Sample_ID)	-0,191	0,158	0,014	0,409	0,079	0,261
Firmicutes	*Clostridia*	Marvinbryantia	FT_cycle	(1 | Sample_ID)	-0,191	0,093	0,118	0,118	0,309	0,255
Bacteroidetes	*Bacteroidia*	Parabacteroides	FT_cycle	(1 | Sample_ID)	0,252	0,196	-0,054	-0,169	-0,215	-0,310
Firmicutes	*Clostridia*	Ruminococcaceae UCG-003	FT_cycle	(1 | Sample_ID)	0,048	-0,048	0,030	-0,032	-0,055	-0,535
Firmicutes	*Clostridia*	Subdoligranulum	FT_cycle	(1 | Sample_ID)	-0,143	0,105	0,094	0,644	-0,004	0,106
Firmicutes	*Clostridia*	unknown_Gen Clostridiales vadinBB60 gr. fam.	FT_cycle	(1 | Sample_ID)	-0,176	-0,215	-0,153	0,022	-0,278	-0,111
