# Supplementary material for: Effects of repeated freeze and thaw cycles on the stability of faecal microbiome composition
Source: Sci Rep. 2026 Feb 19;16:9880. doi: 10.1038/s41598-026-39939-w (PMC13018280; doi:10.1038/s41598-026-39939-w)
Supplement: Supplementary file 2 — Supplementary Material 2 [file 41598_2026_39939_MOESM2_ESM.pdf]

Additional Information

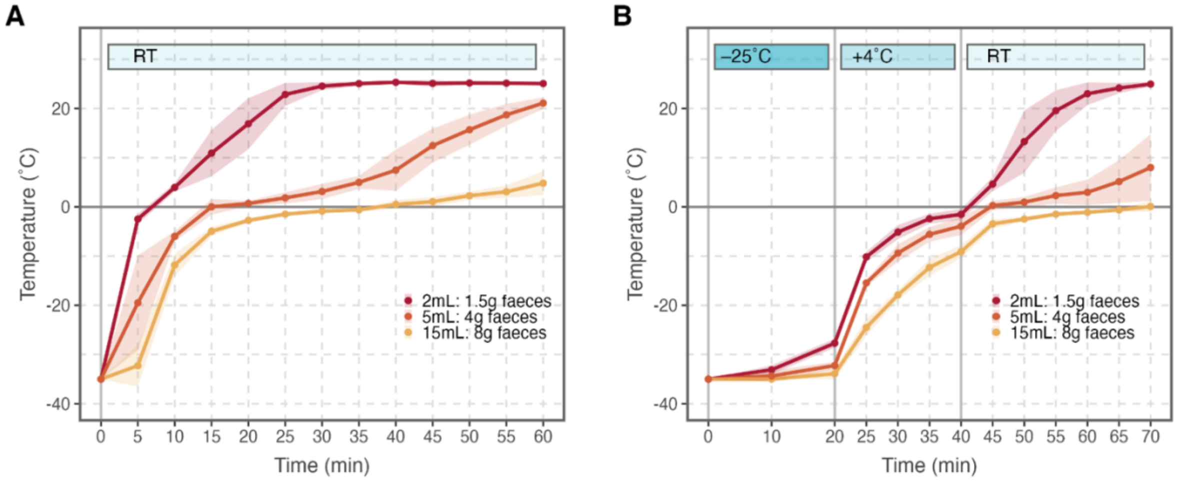

Supplementary Figure 1 – Time changes in temperature of faecal samples using

Temperature changes using a fast (A) or slow (B) thawing process for faecal samples that are stored in 3 different tubes, of 2 mL, 5 mL and 15 m L total volume. The top bars in cyan indicates the temperature at which samples were kept at different times of the thawing process. The chart shows the mean temperature (line) and standard deviation (ribbon area) for repeated measurements (n=3).

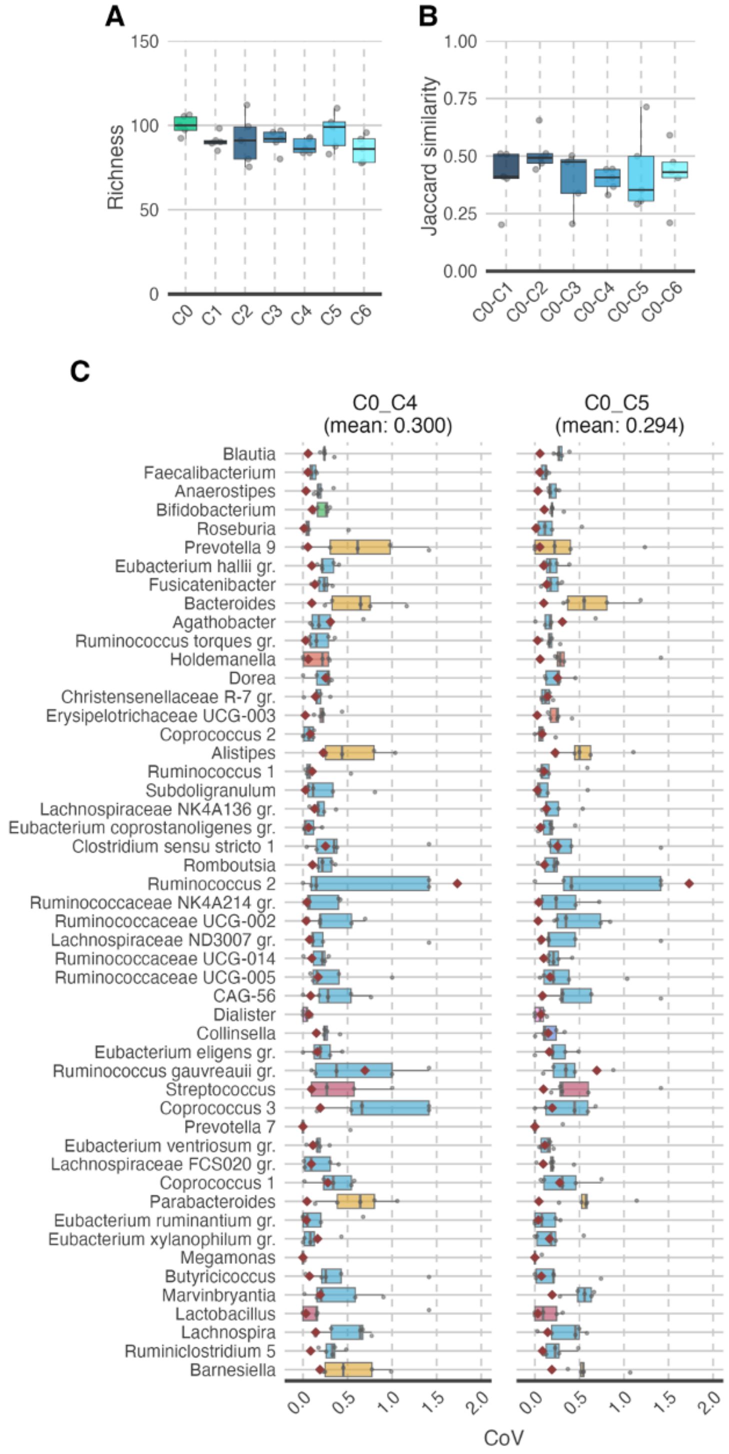

Supplementary Figure 2

A – Genus-level richness at each of the thawing cycle, C0 to C6. **B** – Jaccard beta-diversity metric; calculated comparing the C0 (fresh sample) to each of the thawing cycle, C1 to C6.

C – Coefficient of variation (CoV) for the top 50 most abundant genera (sorted by abundance) in the gut microbiome of the entire cohort. The comparison between Co and FT cycle C4 and C5 are shows. The red dots are control values, CoV calculated from set of quality control samples (n≥3).

Box plot – line, mean; box, delimits 25th and 75th percentile; whiskers, 1.5 interquartile; points, individual values.

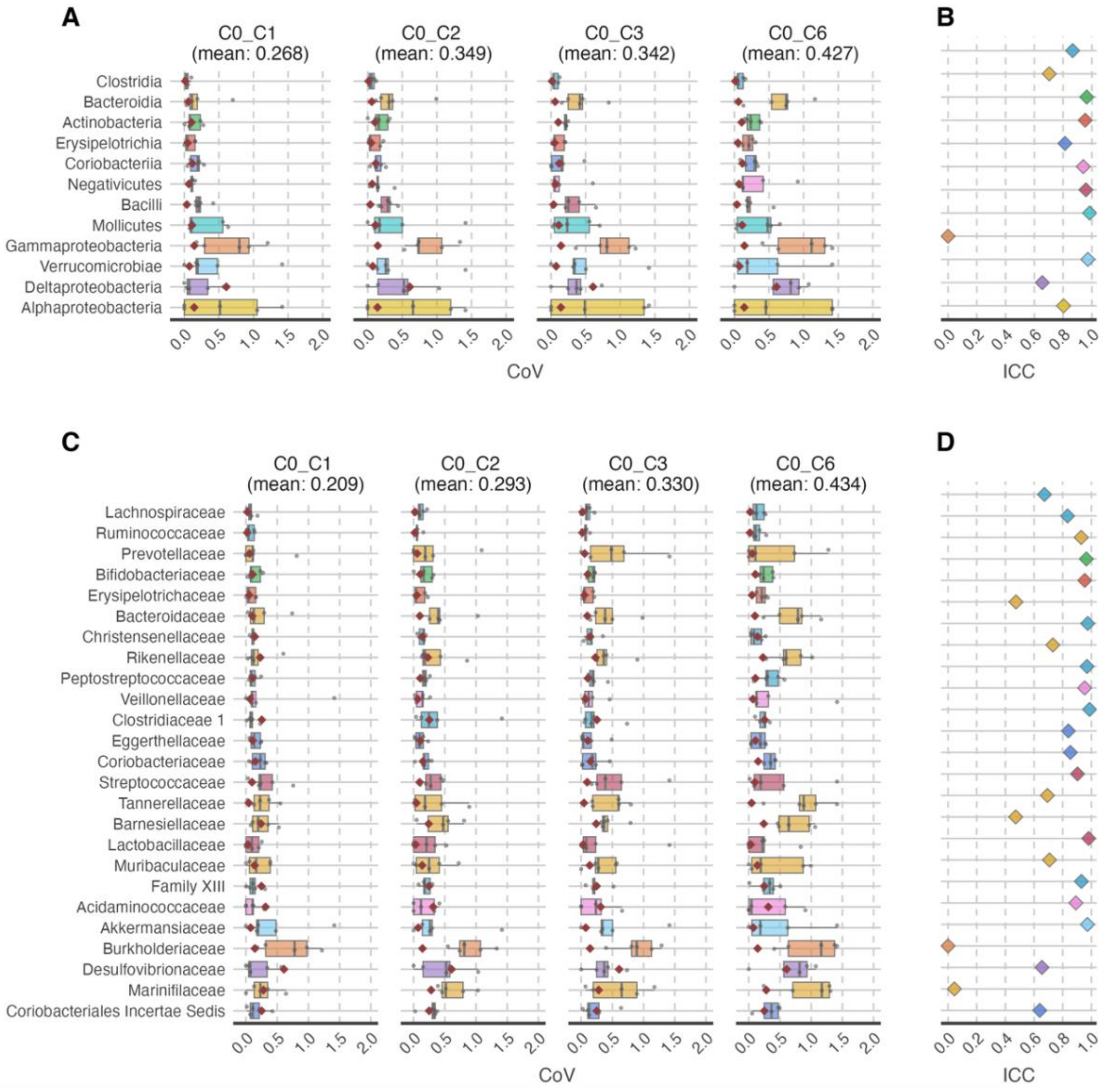

Supplementary Figure 3

A and **B** – Coefficient of variation (CoV) and interclass correlation coefficient (ICC) for the 12 most abundant classes (sorted by abundance) in the gut microbiome. The red dots are CoVs calculated quality control samples (n≥3).

C and **D** –CoV and ICC for the 25 most abundant families (sorted by abundance) in the gut microbiome. The red dots are CoVs calculated quality control samples (n≥3).

Box plot – line, mean; box, delimits 25th and 75th percentile; whiskers, 1.5 interquartile; points, individual values.

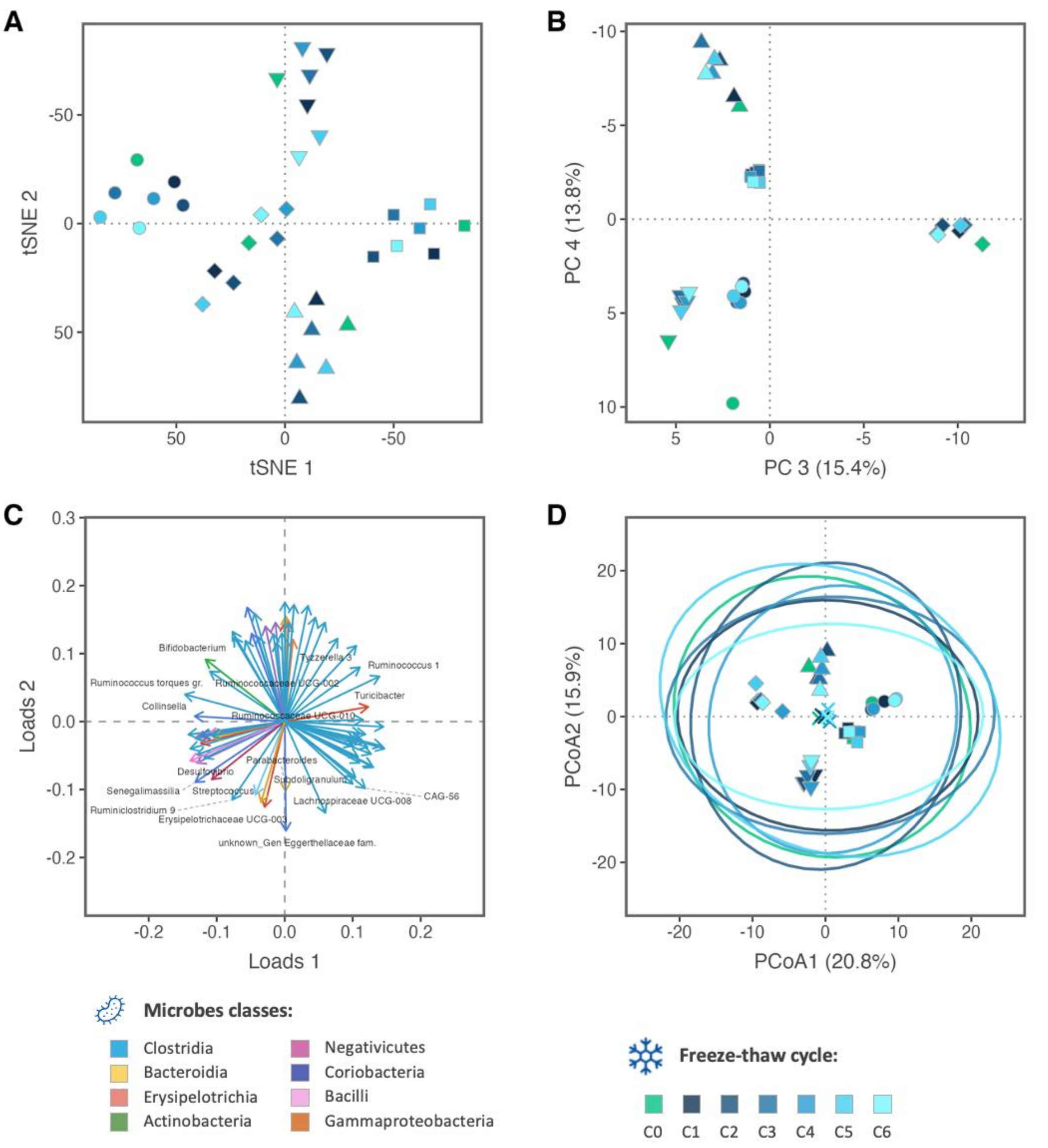

Supplementary Figure 4

A – t-distributed stochastic neighbour embedding (tSNE) clustering of the cohort (perplex = 10).

B–PCA scores for the 3rd and 4th components (variance explained is shown in parenthesis).

C – Loading for components 1 and 2 for the PCA showed in Figure 2A. Arrows colour correspond to microbial classes (see legend)

D – Principal Coordinate Analysis (PCoA) with Bray-Curtis dissimilarity; PERMANOVA test showed no difference in the FT cycles ($p = 0.225$). Sample of different individuals are identified by shape of points, and colours of points and circles indicate the different FT cycles (see lower right legend).
